# High Level of Soluble HLA-G in the Female Genital Tract of Beninese Commercial Sex Workers Is Associated with HIV-1 Infection

**DOI:** 10.1371/journal.pone.0025185

**Published:** 2011-09-23

**Authors:** Valérie Thibodeau, Julie Lajoie, Annie-Claude Labbé, Marcel D. Zannou, Keith R. Fowke, Michel Alary, Johanne Poudrier, Michel Roger

**Affiliations:** 1 Laboratoire d'immunogénétique, Centre de Recherche du Centre Hospitalier de l'Université de Montréal (CRCHUM), Montréal, Canada; 2 Département de Microbiologie et Immunologie de l'Université de Montréal, Montréal, Canada; 3 Department of Medical Microbiology University of Manitoba, Winnipeg, Canada; 4 Département de Microbiologie Médicale, Hôpital Maisonneuve-Rosemont, Montréal, Canada; 5 Centre National Hospitalier Universitaire Hubert K. Maga, Université d'Abomey Calavi, Cotonou, Bénin; 6 Unité de Recherche en Santé des Populations, Centre hospitalier affilié universitaire de Québec and Université Laval, Québec, Canada; 7 Département de médecine sociale et préventive, Université Laval, Québec, Canada; South Texas Veterans Health Care System, United States of America

## Abstract

**Background:**

Most HIV infections are transmitted across mucosal epithelium. Understanding the role of innate and specific mucosal immunity in susceptibility or protection against HIV infection, as well as the effect of HIV infection on mucosal immunity, are of fundamental importance. HLA-G is a powerful modulator of the immune response. The aim of this study was to investigate whether soluble HLA-G (sHLA-G) expression in the female genital tract is associated with HIV-1 infection.

**Methods and Findings:**

Genital levels of sHLA-G were determined in 52 HIV-1-uninfected and 44 antiretroviral naïve HIV-1-infected female commercial sex workers (CSWs), as well as 71 HIV-1-uninfected non-CSW women at low risk of exposure, recruited in Cotonou, Benin. HIV-1-infected CSWs had higher genital levels of sHLA-G compared with those in both the HIV-1-uninfected CSW (P = 0.009) and non-CSW groups (P = 0.0006). The presence of bacterial vaginosis (P = 0.008), and HLA-G*01:01:02 genotype (P = 0.002) were associated with higher genital levels of sHLA-G in the HIV-1-infected CSWs, whereas the HLA-G*01:04:04 genotype was also associated with higher genital level of sHLA-G in the overall population (P = 0.038). When adjustment was made for all significant variables, the increased expression of sHLA-G in the genital mucosa remained significantly associated with both HIV-1 infection (P = 0.02) and bacterial vaginosis (P = 0.03).

**Conclusion:**

This study demonstrates that high level of sHLA-G in the genital mucosa is independently associated with both HIV-1 infection and bacterial vaginosis.

## Introduction

HIV vaccines and microbicides hold promise for preventing the acquisition of HIV-1 infection [1], [2] but successful design of such agents requires a clear understanding of the mechanisms of HIV-1 transmission at the initial site of infection [3]. Most HIV-1 infections occur during heterosexual intercourse, and women are more likely to become infected than men [4]. Initial exposure to HIV-1 during sexual transmission occurs in the genital tract; however, little is known about HIV-1-specific immune responses at this site, as well as the effect of HIV-1 on mucosal immunity.

Human leukocyte antigen (HLA)-G is a non-classical major histocompatibility class I protein, characterised by limited polymorphism and tissue-restricted distribution. HLA-G is expressed as membrane-bound (HLA-G1, -G2, -G3 and -G4) and soluble (HLA-G5, -G6, -G7) isoforms as a result of alternative splicing [5]. The major isoforms present in the plasma are soluble HLA-G (sHLA-G)-1 and -G5 which are generated by shedding or proteolytic cleavage of membrane-bound HLA-G1 isoform and by secretion of a soluble form, respectively. Under physiological conditions, sHLA-G levels correlate with gender and HLA-G genetic polymorphisms. The level of sHLA-G is higher in women than in men [6]. Healthy individuals carrying the HLA-G*01:01:03 and HLA-G*0105N alleles have lower plasma sHLA-G levels than subjects carrying the more frequent HLA-G*01:01:01 allele. In addition, individuals with the latter allele have lower plasma sHLA-G levels than those with the HLA-G*01:04 allele. Polymorphisms in the 3′-untranslated region (3′UTR) can also affect the production of HLA-G molecules. The presence of a 14-bp sequence insertion in HLA-G 3′UTR has been associated with lower levels of sHLA-G in serum of healthy subjects [7]–[9]. HLA-G expression can be induced during pregnancy [10], antiretroviral (ART) therapy [11], [12] and in pathological conditions such as autoimmune diseases, cancers, transplantations, and viral infections [13]. HLA-G molecules inhibit the activity and mediate apoptosis of natural killer (NK) cells and cytotoxic CD8^+^ T cells [14]–[17], as well as CD4^+^ T cell proliferation [18] and induce tolerogenic dendritic cells (DC) and regulatory T cells [19]–[22].

The immunosuppressive properties of HLA-G might contribute to the susceptibility to HIV-1 infection. Recent studies have shown that HLA-G polymorphisms are associated with altered risks of heterosexual acquisition [23]–[25] and vertical transmission [26], [27] of HIV-1. Plasma sHLA-G expression, at the protein level, was recently associated with increased risk of HIV-1 infection and more rapid disease progression [19], [28], [29]. However, initial exposure to HIV-1 during sexual transmission occurs in the female genital tract and no data are available on the possible association between genital HLA-G expression and susceptibility to HIV-1 infection. We have therefore measured the genital levels of sHLA-G in HIV-1-infected and HIV-1-uninfected female commercial sex workers (CSWs), as well as HIV-1-uninfected non-CSW women at low risk for exposure to investigate whether sHLA-G expression is associated with HIV-1 infection.

## Methods

### Study population

Female CSWs were enrolled through a dedicated sex worker clinic in Cotonou, Benin and were divided into two groups: HIV-1-uninfected CSWs (n = 52) and ART-naïve HIV-1-infected CSWs (n = 44). The HIV-1-uninfected non-CSW control subjects at low risk for exposure (n = 71) were enrolled from a general health clinic in Cotonou. Women were invited to participate in the study as they attended clinics. Women were excluded from the study if <18 years old, menstruating, or pregnant. At enrolment, participants were asked to answer a questionnaire about demographic information, sexual behaviour, duration of prostitution, number of sex partners, condom use, vaginal douching practices, and reproductive history. Each participant underwent a genital examination by a physician. Vaginal specimens were obtained for diagnosis of candidiasis and bacterial vaginosis by microscopic examination. Endocervical swabs were obtained to test for *Neisseria gonorrhoeae* and *Chlamydia trachomatis* infection using BD ProbeTec ET system (Strand Displacement Assay, Becton Dickinson, Heidelberg, Germany). Peripheral blood was taken for HIV, HLA-G and CCR5 genotype analyses. Plasma and serum were kept frozen at - 80°C until use. HIV-1 positivity was defined by the presence of HIV-1 antibodies tested with Vironostika HIV Uni-Form II Ag/Ab (Organon Teknika, Boxtel, The Netherlands). Non-reactive samples were considered HIV-seronegative, whereas reactive samples were tested with Genie II HIV-1/HIV-2 (Bio-Rad, Hercules, CA). Genie II dually reactive samples (to HIV-1 and HIV-2) and discordant samples (Vironostika reactive/Genie II non-reactive) were further tested by INNO-LIA HIV I/II Score (Innogenetics NV, Technologiepark 6, Gent, Belgium). Viral loads were determined in the plasma of all HIV-1 infected CSWs using VERSANT HIV-1 RNA 3.0 Assay (bDNA) (Siemens Medical Solutions Diagnostics, Tarrytown NY). DNA samples were genotyped for the CCR5 32-bp deletion allele and all women were found to be homozygous for the wild-type allele.

### Mucosal sample collection and preparation

Cervicovaginal lavage (CVL) samples were obtained from all study participants by a physician, using a 10-ml syringe filled with sterile phosphate-buffered solution and aimed directly into the cervical os. CVL fluids were then collected, transferred immediately into 20 ml of RPMI-1640, kept on ice, and processed within 1 hour. CVL samples were centrifuged at 1500 r.p.m. for 10 min to remove cells and debris, and supernatants were stored at −80°C until shipped on dry ice to Montréal, Canada. CVL samples were concentrated with Amicon Ultra-15 5 kDa (Millipore, Billerica MA) prior to sHLA-G measurement.

### Soluble HLA-G measurements and HLA-G genotyping

sHLA-G CVL levels were measured using the Human sHLA-G Immunoassay kit (Alexis Biochemicals, San Diego, CA, USA), which allows simultaneous detection of HLA-G1 and -G5 soluble proteins without discrimination. The final concentration of sHLA-G in the CVL sample was determined as follows: concentration obtained with the sHLA-G Elisa assay (units per ml)/(CVL concentration factor)×total CVL volume prior to concentration. HLA-G alleles were determined by direct DNA sequencing analysis of the nucleotide regions encompassing HLA-G exons 2–4 and using purified DNA from blood samples as described previously [30]. HLA-G 3-UTR polymorphisms were determined according to the protocol previously described by [31].

### Statistical analysis

Statistical analysis was performed using the GraphPad PRISM 5.0 for Windows (GraphPad Software, San Diego, CA). One-way analysis of variance and Chi-square tests were used to assess the significance of the associations between continuous and categorical variables across all study groups. Comparisons of continuous and categorical variables between two groups were assessed by the Mann-Whitney *U* and Chi-square or Fisher exact tests, respectively. Spearman's rank test was used to determine correlations between continuous variables. Multiple logistic regression analysis was used to define independent predictors identified as significant in the crude analysis. Odds ratio (OR) and 95% confidence interval (CI) were calculated with the exact method. Differences were considered significant at P≤0.05 or P≤0.015 when comparing two or three groups, respectively.

### Ethics statement

Written informed consent was obtained from all subjects who participated in the study and the investigation reported in this paper was approved by the Comité National Provisoire d'Éthique de la Recherche en Santé in Cotonou and the CHUM Research Ethics Committee.

## Results

Sociodemographic and clinical characteristics of the study population are described in Table 1. These data were collected to address the issue of confounding variables for risk of HIV-1 infection. The three groups were similar with respect to age, days from last menses, vaginal douching, and the presence of vaginal candidiasis. The HIV-1-infected CSWs were more likely to have a bacterial vaginosis (P = 0.003) than the HIV-1-uninfected non-CSWs. The HIV-1-unifected non-CSWs, were less likely to have *Chlamydia trachomatis* or *Neisseria gonorrhoeae* genital infections than the HIV-1-uninfected (P = 0.027) and HIV-1-infected (P = 0.022) CSW groups. The average number of clients was higher in HIV-1-uninfected CSWs than in HIV-1-infected CSWs (P = 0.044), whereas the duration of sex work, and condom use were equivalent between the two CSW groups.

**Table 1:** Distribution of demographic, sexual behaviour and genital tract infection characteristics in HIV-1-uninfected CSWs, HIV-1-infected CSWs, and HIV-1-uninfected non-CSW women.

	HIV-1-uninfected	HIV-1-infected	HIV-1-uninfected	p
	CSWs	CSWs	non-CSWs	value^a^
	N = 52	N = 44	N = 71	
Age, mean (SD), years	34.4 (12.3)	34 (8.7)	32.6 (9.4)	NS
Duration of sex work, mean (SD), years	4.3 (3.2)	4.1 (2.6)	NA	NS
Number of clients last week, mean (SD)	17.1 (14.0)	11.3 (11.0)	NA	0.044
Days since last menses, mean (SD)	17.1 (13.0)	18.2 (15.4)	19.1 (12.3)	NS
Vaginal douching	50/52 (96%)	42/43 (98%)	65/71 (93%)	NS
Condom always used with clients past month	39/52 (75%)	22/39 (56%)	NA	NS
Bacterial vaginosis	33/51 (65%)	34/43 (79%)	36/71 (51%)	0.009^b^
Candidiasis	4/51 (8%)	5/44 (11%)	15/71 (21%)	NS
NG and/or CT infections	7/46 (15%)	6/39 (15%)	2/72 (3%)	0.029^c^

CSW, commercial sex worker; HIV-1, human immunodeficiency virus type 1; N: number of participants; NA: non applicable; NG/CT: *Neisseria gonorrhoeae*/*Chlamydia trachomatis*, NS: nonsignificant; SD, standard deviation.

a P-values for the comparison across all groups were calculated with one-way ANOVA analysis of variance for the age and days since last menses; Mann-Whitney U test for the duration of sex work and average number of clients; Chi-square test for vaginal douching, condom use, bacterial vaginosis, candidiasis, and NG/CT infections.

b P = 0.125 for the comparison between HIV-1-uninfected CSWs and HIV-1-infected CSWs, P = 0.105 for the comparison between HIV-1-uninfected CSWs and HIV-1-uninfected non-CSWs, and P = 0.003 for the comparison between HIV-1-infected CSWs and HIV-1-uninfected non-CSWs as determined by Chi-square test.

c P = 0.987 for the comparison between HIV-1-uninfected CSWs and HIV-1-infected CSWs, P = 0.027 for the comparison between HIV-1-uninfected CSWs and HIV-1-uninfected non-CSWs, and P = 0.022 for the comparison between HIV-1-infected CSWs and HIV-1-uninfected non-CSWs as determined by Fisher exact test.

**Figure 1:**
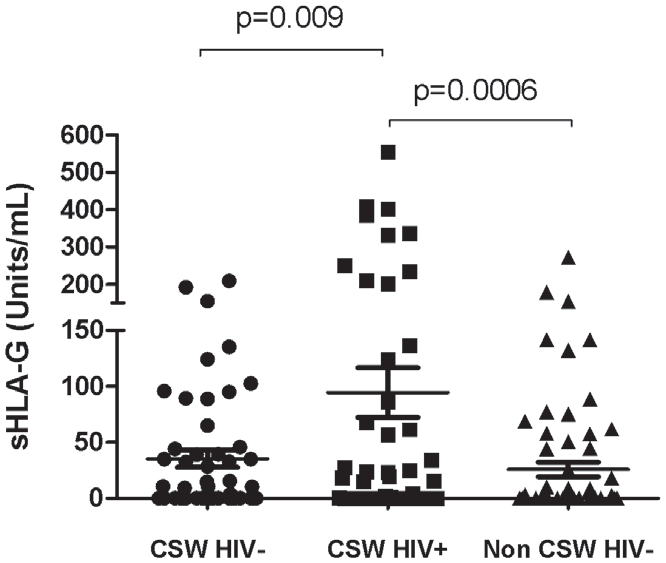
Mean genital soluble HLA-G levels according to the study groups. Statistical significance of differences in the genital levels were evaluated with the Mann-Whitney *U* test. CSW, commercial sex worker; HIV-1, human immunodeficiency virus type 1.

HIV-1-infected CSWs had significantly higher levels of sHLA-G in their CVL samples (94±145 units/ml) than did the HIV-1-uninfected CSWs (35±53 units/ml; P = 0.009) and the HIV-1-uninfected non-CSW women (26±53 units/ml; P = 0.0006) (Figure 1). There was no significant correlation between the HIV-1 plasma viral load and the sHLA-G level in the CVLs of the HIV-1-infected CSWs (r^2^ = −0.162, P = 0.344).

Since sHLA-G expression has been associated with HLA-G polymorphism [7]–[9], we looked at the distribution of sHLA-G levels, either between study groups or in the total population, according to the HLA-G genetic variants (Table 2). The HLA-G*01:01:02 genotype, in the heterozygous or homozygous states, was associated with increased expression of genital sHLA-G in HIV-1-infected CSWs compared with those in both the HIV-1-uninfected CSW (P = 0.051) and non-CSW (P = 0.002) groups. In the overall population, women carrying the HLA-G*01:04:04 heterozygous or homozygous genotypes expressed the highest levels of genital sHLA-G molecules when compared with those expressed by women harbouring other genotypes (P = 0.038). However, there was no significant association between HLA-G alleles and sHLA-G levels within the three groups taken separately. Because HLA-G polymorphism can also be associated with HIV-1 infection [23]–[27], we looked at the distribution of the HLA-G genetic variants among the study groups (Table 2) and found no significant association between HLA-G alleles and HIV-1 infection (data not shown). The presence of bacterial vaginosis could potentially affect the genital level of sHLA-G molecules and since the rate of bacterial vaginosis was significantly higher in the HIV-1-infected CSWs (Table 1), we investigated the possible correlation between sHLA-G levels and the presence of bacterial vaginosis (Table S1). We found that the expression of sHLA-G in genital samples was significantly associated with bacterial vaginosis among the HIV-1-infected CSWs (P = 0.035).

**Table 2:** Genital soluble HLA-G levels in HIV-1-uninfected CSWs, HIV-1-infected CSWs, and HIV-1- uninfected non-CSW women according to the HLA-G gene polymorphism.

HLA-G	HIV-1- uninfected		HIV-1-infected		HIV-1 uninfected		P-value^a^	Total population		P-value^b^
	CSWs		CSWs		non-CSWs					
allele^c^	N	Levels	N	Levels	N	Levels		N	Levels	
01:01:01	34	59.4 (148)	20	113 (230)	33	48.0 (86)	NS	87	67.0 (152)	NS
01:01:02	15	64.5 (203)	14	191 (259)	15	3.9 (12)	0.034^d^	44	84.0 (200)	NS
01:03	11	47.8 (73)	11	59.4 (109)	8	12.2 (27)	NS	30	42.6 (81)	NS
01:04:01	4	48.2 (96)	7	171 (228)	14	18.0 (49)	NS	25	65.6 (142)	NS
01:04:04	20	118 (248)	19	118 (165)	12	31.8 (59)	NS	51	97.6 (188)	0.038
0105N	8	30.7 (32)	5	67.4 (151)	10	14.1 (42)	NS	23	31.5 (75)	NS
3′UTR SNP^e^										
3777 (c/c)	12	81.8 (228)	5	61.6 (107)	13	6.5 (21)	NS	30	45.8 (151)	NS
3952 (a/a)	27	86.7 (217)	11	64.6 (134)	27	39.1 (77)	NS	65	64.0 (158)	NS
14-bp (I/I)	6	16.4 (36)	7	97.7 (135)	6	0.45 (1.1)	NS	19	41.3 (92)	NS

CSW, commercial sex worker; HIV-1, human immunodeficiency virus type 1; I, insertion,

N, number of participants; NS, nonsignificant; SD, standard deviation; SNP, single nucleotide polymorphism; UTR, untranslated region.

Data are mean (SD).

a P-values for the comparison between all groups were calculated with one-way analysis of variance test.

b P-values were calculated with Mann-Whitney U test.

c Presence of the allele in the homozygous or heterozygous states.

d P = 0.051 for the comparison between HIV-1-uninfected CSWs and HIV-1-infected CSWs, P = 0.153 for the comparison between HIV-1-uninfected CSWs and HIV-1-uninfected non-CSWs and P = 0.002 for the comparison between HIV-1-infected CSWs and HIV-1-uninfected non-CSWs as determined by Mann-Whitney U test.

e Presence of the variants in the homozygous state.

When adjustment was made for all significant variables found in the crude analysis (HIV-1 infection, bacterial vaginosis, HLA-G*01:01:02 and HLA-G*01:04:04 genotypes), the expression of sHLA-G in the genital mucosa remained significantly associated with both HIV-1 infection (OR: 3.0, 95% CI = 1.17–7.53, P = 0.02) and bacterial vaginosis (OR 3.4, 95% CI = 1.10–10.5, P = 0.03).

## Discussion

High level of sHLA-G in the genital mucosa is associated with HIV-1 infection in Beninese CSWs. In the present study, we have carefully controlled for potential confounding factors that could influence HLA-G expression such as gender [6], pregnancy [10], ART therapy [11], [12] and HLA-G polymorphism [7]–[9]. All study participants were ART-naïve nonpregnant women. The HLA-G*01:01:02 and HLA-G*01:04:04 genotypes were significantly associated with sHLA-G expression in the crude analysis but these associations disappeared after adjustment was done for HIV-1 infection. In contrast to previous studies [23]–[25], HLA-G polymorphism was not associated with risk of HIV-1 infection among the Beninese CSWs. The relatively small number of subjects analysed in each groups have limited the power of the present study to reproduce previous findings.

We have previously measured the level of sHLA-G in the blood of these women and found that HIV-infected CSWs had lower plasma levels when compared to HIV-uninfected CSWs and non-CSWs. This is in sharp contrast with that found in the genital mucosa of these women. The discordance in the production of sHLA-G between the two compartments may depend on local factors such as immune cells, micro-organisms and derived products that could affect sHLA-G expression. sHLA-G plays a crucial role in the regulation of both innate and adaptive immunity by modulating the function of DC, NK and T lymphocytes [14]–[22]. These effects depend on interactions of HLA-G molecules with inhibitory receptors expressed on myeloid cells (immunoglobuline-like transcripts (ILT)-4), on myeloid and lymphoid cells (ILT-2) and on NK cells (killing inhibitory receptor (KIR)-2DL4) [32]. The outcome of the immune response may therefore vary according to the specific interactions of sHLA-G with the different types of cells and receptors. Interaction of sHLA-G with ILT-2 receptor on DC and NK cells decreased the release of interferon (INF)-gamma and increased the production of interleukin (IL)-10 and transforming growth factor (TGF)-beta [33], [34]. IL-10 has been shown to induce HLA-G expression [35] and HLA-G can also stimulate IL-10 expression in peripheral blood monocytes [36]. Triggering ILT-4 by sHLA-G induces tolerogenic DC and T regulatory cells [20], [37]. On the other hand, interaction of sHLA-G with KIR2DL4 receptor on peripheral blood monocytes and NK cells promotes the production of pro-inflammatory cytokines and chemokines [36], [38]–[40]. We have previously measured the cytokine and chemokine expression patterns in the genital samples of our study subjects and found that HIV-1-infected CSWs had significantly higher levels of IFN-gamma tumor necrosis factor (TNF)-alpha, monocyte chemotactic protein (MCP-3/CCL7) and monokine induced by IFN-gamma (MIG/CXCL9) compared with those in both the HIV-1-uninfected CSW and non-CSW groups [41], [42]. The same observations were made for IL-1 beta and IL-8 (data unpublished). High level of IL-1 beta and TNF-alpha in the female genital tract has been associated with enhanced HIV-1 shedding at this site [43]. The inflammatory response observed in the genital mucosa of HIV-1-infected women may promote the recruitment, differentiation and activation of immune cells, which act as targets favouring viral replication and viral dissemination at the initial site of infection. As to whether sHLA-G is directly involved in the induction of such mucosal inflammation via its interaction with KIRD2L4 on monocytes and NK cells in the female genital tract remains to be confirmed. Although the genital mucosa levels of sHLA-G correlate significantly with those of the cytokines and chemokines in the HIV-1-uninfected groups, these correlations were not significant in the HIV-1-infected CSW group (Tables S2 and S3). Thus, in the absence of HIV-1, genital levels of the immunosuppressive sHLA-G molecules and pro-inflammatory cytokines and chemokines are low and correlate to maintain mucosal homeostasis. Conversely, in the presence of HIV-1, there is an aberrant and independent production of both factors in the female genital tract that may reflect a viral strategy of immune piracy, allowing for the simultaneous production of chemokines/cytokines to recruit and activate HIV-1 target cells and sHLA-G to induce immune tolerance towards HIV-1.

Interestingly, the increased level of sHLA-G in genital samples was also significantly associated with the presence of bacterial vaginosis. Although HIV-1-infected CSWs had higher levels of sHLA-G and were more likely to have a bacterial vaginosis than the HIV-1-uninfected non-CSWs, the association between sHLA-G levels and bacterial vaginosis remained significant after adjusting for HIV-infection. This suggests that genital sHLA-G level is independently associated with both bacterial vaginosis and HIV-1 infection. Bacterial vaginosis is an established risk factor for HIV infection [44], [45]. It has been suggested that bacterial vaginosis increases risk of HIV infection by inducing a clinical or subclinical mucosal inflammatory response, recruiting target cells and breaching of intact cervico-vaginal mucosa [46]. Indeed, bacterial vaginosis has been associated with increased levels of IL-1 beta, IL-6, IL-8, IL-10 and TNF-alpha, RANTES (CCL5), macrophage inflammatory protein (MIP-1 alpha/CCL3) and MIP-1 beta (CCL4) in genital samples [47]–[49]. However, bacterial vaginosis was not associated with the production of these cytokines and chemokines in the genital tract of the Beninese women (Tables S4 and S5).

Altogether, these results suggest that in the context of HIV-1 infection, sHLA-G expression in the female genital tract is a complex process modulated by many factors such as HIV-1, bacterial vaginosis HLA-G genotypes, and cytokine/chemokine expression patterns, which may all contribute to an immunological environment promoting viral replication and escape from the mucosal immune response.

## Supporting Information

Table S1 sHLA-G genital levels according to the presence or absence of vaginosis in HIV-1-uninfected CSWs, HIV-1-infected CSWs, and HIV-1- uninfected non-CSW control subjects.
(DOC)

Table S2 Spearman's correlations between soluble HLA-G and cytokine genital levels in HIV-1-uninfected CSWs, HIV-1-infected CSWs, and HIV-1-uninfected non-CSW women.
(DOC)

Table S3 Spearman's correlations between soluble HLA-G and chemokine genital levels in HIV-1-uninfected CSWs, HIV-1-infected CSWs, and HIV-1- uninfected non-CSW women.
(DOC)

Table S4 Cytokine genital levels according to the presence or absence of bacterial vaginosis in HIV-1-uninfected CSWs, HIV-1-infected CSWs, and HIV-1- uninfected non-CSW women.
(DOC)

Table S5 Chemokine genital levels according to the presence or absence of bacterial vaginosis in HIV-1-uninfected CSWs, HIV-1-infected CSWs, and HIV-1- uninfected non-CSW women.
(DOC)
