# Supplementary material for: High Level of Soluble HLA-G in the Female Genital Tract of Beninese Commercial Sex Workers Is Associated with HIV-1 Infection
Source: PLoS One. 2011 Sep 23;6(9):e25185. doi: 10.1371/journal.pone.0025185 (PMC3179477; doi:10.1371/journal.pone.0025185)
Supplement: Table S1 — sHLA-G genital levels according to the presence or absence of vaginosis in HIV-1-uninfected CSWs, HIV-1-infected CSWs, and HIV-1- uninfected non-CSW control subjects. (DOC) [file pone.0025185.s001.doc]

**Table S1** sHLA-G genital levels according to the presence or absence of vaginosis in HIV-1-uninfected CSWs, HIV-1-infected CSWs, and HIV-1- uninfected non-CSW control subjects.

|  | HIV-1-uninfected CSWs | | | | | HIV-1-infected CSWs | | | | | HIV-1-uninfected non-CSWs | | | | |
| --- | --- | --- | --- | --- | --- | --- | --- | --- | --- | --- | --- | --- | --- | --- | --- |
|  | Bacterial Vaginosis | | | | | Bacterial Vaginosis | | | | | Bacterial Vaginosis | | | | |
|  | N | **+** | N | **-** | P-value^a^ | N | **+** | N | **-** | P-value^a^ | N | **+** | N | **-** | P-value^a^ |
| sHLA-G | 33 | 81 (198) | 18 | 42 (63) | 0.460 | 34 | 118 (155) | 9 | 110 (309) | 0.035 | 70 | 80 (128) | 44 | 55 (158) | 0.007 |

CSW, commercial sex worker; HIV-1, human immunodeficiency virus type 1; N, number of participants;

Data are mean (SD).

^a^ P-values were calculated with Mann-Whitney *U* test.
