# Supplementary material for: High Level of Soluble HLA-G in the Female Genital Tract of Beninese Commercial Sex Workers Is Associated with HIV-1 Infection
Source: PLoS One. 2011 Sep 23;6(9):e25185. doi: 10.1371/journal.pone.0025185 (PMC3179477; doi:10.1371/journal.pone.0025185)
Supplement: Table S2 — Spearman's correlations between soluble HLA-G and cytokine genital levels in HIV-1-uninfected CSWs, HIV-1-infected CSWs, and HIV-1-uninfected non-CSW women. (DOC) [file pone.0025185.s002.doc]

**Table S2** Spearman’s correlations between soluble HLA-G and cytokine genital levels in HIV-1-uninfected CSWs, HIV-1-infected CSWs, and HIV-1-uninfected non-CSW women.

|  | HIV-1-uninfected | | | HIV-1-infected | | | HIV-1-uninfected | | |
| --- | --- | --- | --- | --- | --- | --- | --- | --- | --- |
|  | CSWs | | | CSWs | | | non-CSWs | | |
|  | N |  |  | N |  |  | N |  |  |
| IL-1 beta | 49 | r^2^ | 0.325 | 41 | r^2^ | 0.067 | 64 | r^2^ | 0.364 |
|  |  | p value | 0.023 |  | p value | 0.677 |  | p value | 0.003 |
| IL-6 | 49 | r^2^ | 0.400 | 42 | r^2^ | 0.221 | 67 | r^2^ | 0.477 |
|  |  | p value | 0.004 |  | p value | 0.160 |  | p value | <0.0001 |
| IL-8 | 47 | r^2^ | 0.208 | 43 | r^2^ | 0.046 | 63 | r^2^ | -0.042 |
|  |  | p value | 0.016 |  | p value | 0.769 |  | p value | 0.746 |
| IL-10 | 49 | r^2^ | 0.305 | 41 | r^2^ | 0.056 | 65 | r^2^ | 0.492 |
|  |  | p value | 0.033 |  | p value | 0.728 |  | p value | <0.0001 |
| TNF-alpha | 49 | r^2^ | 0.291 | 42 | r^2^ | 0.072 | 66 | r^2^ | 0.323 |
|  |  | p value | 0.043 |  | p value | 0.648 |  | p value | 0.008 |
| IFN-gamma | 48 | r^2^ | 0.349 | 42 | r^2^ | 0.013 | 64 | r^2^ | 0.262 |
|  |  | p value | 0.015 |  | p value | 0.937 |  | p value | 0.037 |

CSW, commercial sex worker; HIV-1, human immunodeficiency virus type 1; N: number of participants.
