# Supplementary material for: High Level of Soluble HLA-G in the Female Genital Tract of Beninese Commercial Sex Workers Is Associated with HIV-1 Infection
Source: PLoS One. 2011 Sep 23;6(9):e25185. doi: 10.1371/journal.pone.0025185 (PMC3179477; doi:10.1371/journal.pone.0025185)
Supplement: Table S3 — Spearman's correlations between soluble HLA-G and chemokine genital levels in HIV-1-uninfected CSWs, HIV-1-infected CSWs, and HIV-1- uninfected non-CSW women. (DOC) [file pone.0025185.s003.doc]

**Table S3** Spearman’s correlations between soluble HLA-G and chemokine genital levels in HIV-1-uninfected CSWs, HIV-1-infected CSWs, and HIV-1- uninfected non-CSW women.

|  | HIV-1-uninfected | | | HIV-1-infected | | | HIV-1-uninfected | | |
| --- | --- | --- | --- | --- | --- | --- | --- | --- | --- |
|  | CSWs | | | CSWs | | | non-CSW controls | | |
|  | N |  |  | N |  |  | N |  |  |
| MCP-1 | 49 | r^2^ | 0.411 | 43 | r^2^ | 0.132 | 65 | r^2^ | 0.149 |
|  |  | p-value | 0.003 |  | p-value | 0.397 |  | p-value | 0.235 |
| MCP-3 | 50 | r^2^ | 0.386 | 42 | r^2^ | -0.048 | 64 | r^2^ | 0.271 |
|  |  | p-value | 0.006 |  | p-value | 0.761 |  | p-value | 0.030 |
| MIG | 49 | r^2^ | 0.197 | 42 | r^2^ | -0.058 | 65 | r^2^ | 0.206 |
|  |  | p-value | 0.175 |  | p-value | 0.715 |  | p-value | 0.100 |
| MIP-1 alpha | 49 | r^2^ | 0.239 | 42 | r^2^ | -0.045 | 65 | r^2^ | 0.185 |
|  |  | p-value | 0.100 |  | p-value | 0.775 |  | p-value | 0.137 |
| MIP-1 beta | 49 | r^2^ | 0.327 | 42 | r^2^ | 0.079 | 66 | r^2^ | 0.430 |
|  |  | p-value | 0.022 |  | p-value | 0.621 |  | p-value | 0.0003 |
| RANTES | 50 | r^2^ | 0.480 | 41 | r^2^ | -0.063 | 65 | r^2^ | 0.312 |
|  |  | p-value | 0.0004 |  | p-value | 0.696 |  | p-value | 0.011 |

CSW, commercial sex worker; HIV-1, human immunodeficiency virus type 1; N: number of participants.
