# Supplementary material for: High Level of Soluble HLA-G in the Female Genital Tract of Beninese Commercial Sex Workers Is Associated with HIV-1 Infection
Source: PLoS One. 2011 Sep 23;6(9):e25185. doi: 10.1371/journal.pone.0025185 (PMC3179477; doi:10.1371/journal.pone.0025185)
Supplement: Table S4 — Cytokine genital levels according to the presence or absence of bacterial vaginosis in HIV-1-uninfected CSWs, HIV-1-infected CSWs, and HIV-1- uninfected non-CSW women. (DOC) [file pone.0025185.s004.doc]

|  | HIV-1-uninfected CSWs | | | | HIV-1-infected CSWs | | | | HIV-1-uninfected non-CSW controls | | | |
| --- | --- | --- | --- | --- | --- | --- | --- | --- | --- | --- | --- | --- |
|  | Bacterial vaginosis | | | | Bacterial vaginosis | | | | Bacterial vaginosis | | | |
|  | N | **+** | **-** | P-value^a^ | N | **+** | **-** | P-value^a^ | N | **+** | **-** | P-value^a^ |
| IL-1 beta | 56 | 271 (577) | 57.5 (65.1) | 0.361 | 45 | 224 (455) | 157 (222) | 0.632 | 68 | 151 (226) | 61.6 (95.5) | 0.067 |
| IL-6 | 56 | 51.5 (173) | 83.3 (93.6) | 0.107 | 50 | 85.8 (140) | 28.1 (27.3) | 0.654 | 70 | 99.1 (182) | 121 (244) | 0.174 |
| IL-8 | 55 | 4392 (9896) | 2130 (2152) | 0.181 | 45 | 5518 (9211) | 20562 (55063) | 0.557 | 69 | 4245 (7650) | 2500 (3748) | 0.890 |
| IL-10 | 56 | 0.93 (1.9) | 1.10 (3.2) | 0.856 | 50 | 0.91 (2.3) | 0.84 (1.1) | 0.262 | 71 | 2.45 (4.0) | 6.10 (27.1) | 0.152 |
| TNF-alpha | 57 | 8.15 (15.6) | 1.50 (3.1) | 0.390 | 50 | 15.0 (26.1) | 7.80 (9.9) | 0.929 | 68 | 5.70 (12.2) | 4.30 (11.8) | 0.108 |
| IFN-gamma | 54 | 1.20 (3.0) | 0.17 (0.7) | 0.548 | 50 | 12.3 (30.4) | 3.49 (6.5) | 0.263 | 69 | 4.00 (11.7) | 2.50 (13.7) | 0.054 |

**Table S4** Cytokine genital levels according to the presence or absence of bacterial vaginosis in HIV-1-uninfected CSWs, HIV-1-infected CSWs, and HIV-1- uninfected non-CSW women.

CSW, commercial sex worker; HIV-1, human immunodeficiency virus type 1; N, number of participants;

Data are mean (SD).

^a^ P-values were calculated with Mann-Whitney U test.
