# Supplementary material for: High Level of Soluble HLA-G in the Female Genital Tract of Beninese Commercial Sex Workers Is Associated with HIV-1 Infection
Source: PLoS One. 2011 Sep 23;6(9):e25185. doi: 10.1371/journal.pone.0025185 (PMC3179477; doi:10.1371/journal.pone.0025185)
Supplement: Table S5 — Chemokine genital levels according to the presence or absence of bacterial vaginosis in HIV-1-uninfected CSWs, HIV-1-infected CSWs, and HIV-1- uninfected non-CSW women. (DOC) [file pone.0025185.s005.doc]

**Table S5** Chemokine genital levels according to the presence or absence of bacterial vaginosis in HIV-1-uninfected CSWs, HIV-1-infected CSWs, and HIV-1- uninfected non-CSW women.

|  | HIV-1-uninfected CSWs | | | | HIV-1-infected CSWs | | | | HIV-1-uninfected non-CSW controls | | | |
| --- | --- | --- | --- | --- | --- | --- | --- | --- | --- | --- | --- | --- |
|  | Bacterial vaginosis | | | | Bacterial vaginosis | | | | Bacterial vaginosis | | | |
|  | N | **+** | **-** | P-value^a^ | N | **+** | **-** | P-value^a^ | N | **+** | **-** | P-value^a^ |
| MCP-1 | 49 | 41.8 (144) | 18.9 (31) | 0.507 | 43 | 56.2 (94) | 44.5 (56) | 0.887 | 67 | 40.7 (95) | 33.1 (86) | 0.858 |
| MCP-3 | 50 | 13.3 (29) | 7.30 (11) | 0.783 | 42 | 13.4 (17) | 14.7 (18) | 0.555 | 67 | 7.00 (10.8) | 4.70 (6.8) | 0.875 |
| MIP-1 alpha | 50 | 2.50 (3.9) | 4.90 (6.8) | 0.078 | 42 | 2.50 (2.9) | 5.50 (6.1) | 0.129 | 67 | 1.4 0(1.6) | 3.60 (9.8) | 0.263 |
| MIP-1 beta | 48 | 55.8 (67) | 142 (122) | 0.007 | 43 | 91.8 (128) | 185 (219) | 0.124 | 67 | 68.3 (92) | 158 (416) | 0.351 |
| RANTES | 50 | 20.0 (41) | 10.4 (19) | 0.696 | 42 | 12.9 (19) | 48.2 (106) | 0.274 | 68 | 6.70 (8.3) | 4.40 (7.5) | 0.124 |

CSW, commercial sex worker; HIV-1, human immunodeficiency virus type 1; N, number of participants;

Data are mean (SD).

^a^ P-values were calculated with Mann-Whitney U test.
